# Carbohydrate-to-carbohydrate interactions between α2,3-linked sialic acids on α2 integrin subunits and asialo-GM1 underlie the bone metastatic behaviour of LNCAP-derivative C4-2B prostate cancer cells

**DOI:** 10.1042/BSR20140096

**Published:** 2014-09-17

**Authors:** Séverine Van Slambrouck, Sophie Groux-Degroote, Marie-Ange Krzewinski-Recchi, Aurélie Cazet, Philippe Delannoy, Wim F. A. Steelant

**Affiliations:** *School of Science, Technology and Engineering Management, St. Thomas University, 16401 NW 37th Avenue, Miami Gardens, FL 33054, USA; †Unité de Glycobiologie Structurale et Fonctionnelle, UMR CNRS No. 8576, Bâtiment C9, Université des Sciences et Technologies de Lille, F-59655 Villeneuve d’Ascq, Lille, France

**Keywords:** bone metastasis, carbohydrate–carbohydrate interaction, collagen type I, glycosphingolipid, integrin, sialylation, ALP, alkaline phosphatase, AsGM1, asialo-GM1, CCI, carbohydrate-to-carbohydrate interaction, CPI, carbohydrate-to-protein interaction, *C*_T_, threshold cycle value, DIG, digoxigenin, ECM, extracellular matrix, FAK, focal adhesion kinase, GSL, glycosphingolipid, IP, immunoprecipitation, MAA, *Maackia amurensis* agglutinin, NBT/BCIP, Nitro Blue Tetrazolium/5-bromo-4-chloroindol-3-yl phosphate, QPCR, quantitative PCR, SNA, *Sambucus nigra* agglutinin, ST, sialyltransferase, TMA, tissue microarray

## Abstract

Complex interplays among proteins, lipids and carbohydrates can alter the phenotype and are suggested to have a crucial role in tumour metastasis. Our previous studies indicated that a complex of the GSLs (glycosphingolipids), AsGM1 (asialo-GM1), which lacks α2,3-linked sialic acid, and α2β1 integrin receptors is responsible for the metastatic behaviour of C4-2B prostate cancer cells. Herein, we identified and addressed the functional significance of changes in sialylation during prostate cancer progression. We observed an increase in α2,3-linked sialic acid residues on α2 subunits of α2β1 integrin receptors, correlating with increased gene expression of α2,3-STs (sialyltransferases), particularly *ST3GAL3*. Cell surface α2,3-sialylation of α2 subunits was required for the integrin α2β1-dependent cell adhesion to collagen type I and the same α2,3-linked sialic acid residues on the integrin receptor were responsible for the interaction with the carbohydrate moiety of AsGM1, explaining the complex formation between AsGM1 and α2β1 integrin receptors. These results provide novel insights into the role of sialic acids in the organization and function of important membrane components in invasion and metastatic processes.

## INTRODUCTION

Integrins are heterodimeric transmembrane receptors that play a role in cellular adhesion and migration. In cancer cells, they also regulate the invasive behaviour, which is ultimately responsible for the formation of metastases. The activity of the integrins is assumed to be controlled by inside-out signalling mechanisms that induce conformational changes, modulating their affinity for the respective ECM (extracellular matrix) ligands [1]. Additionally, GSLs (glycosphingolipids), including gangliosides, common components of the cell membrane, are known to modify integrin-mediated activities due to the interactions of GSLs with integrin glycans. The latter is also suggested to result in the formation of dynamic microdomains, and this through the establishment of (*cis*)-CCIs (carbohydrate-to-carbohydrate interactions) [2]. Moreover, it is well known that cell-surface protein-linked carbohydrates are involved in protein folding, their stability and hence also their activity. In view of the integrins, there is increasing evidence indicating that these carbohydrate structures affect the activity and specificity of cell–matrix interactions, and are thus influencing cell adhesion, migration and tumour malignancy as well [3].

Glycan structures that are expressed on secreted and cell surface glycoproteins and glycolipids, are essential for the regulation of normal cellular functions [4]. Changes in the structure and expression of carbohydrates are recurrent features in cancer cells and often correlate with human cancer progression, metastatic phenotypes and unfavourable prognosis. In general, these glycan structures tend to be more branched and with increased terminal sialylation [2–5]. The addition of these sialic acids is catalysed by a variety of STs (sialyltransferases), with main categories (i) α2,3-STs, encoded by *ST3GAL* genes; (ii) and α2,6-STs by *ST6GAL* genes resulting in α2,3- or α2,6-linked sialic acids, respectively [6]. Although it is apparent from the literature that changes in terminal sialylation are of great importance during malignant transformation and cancer progression, reported data on the specific type of sialyl-linkage and the level of sialylation are very controversial and inconclusive.

Changes in glycosylation may take place on some specific molecules. In the context of adhesion, migration and invasive behaviour, it has been shown that the integrin glycan composition is important for its structure, function and activity. This has been demonstrated for the α5β1 fibronectin-binding integrin receptor. An early study indicated that glycosylation of the α5 and β1 subunits were crucial for the dimerization of these subunits and for their optimal binding to fibronectin [7]. Furthermore, it was demonstrated that sugar remodelling through the expression of GnT-V (*N*-acetylglucosaminyltransferase V) or increased β1,6 branched *N*-glycans, resulting in increased branching of the glycan chain of the β1 integrin subunit, inhibited the organization of α5β1 with F-actin into extended microfilaments in cells when plated onto fibronectin. This also resulted in stimulated cell migration towards fibronectin and invasion through matrigel [8,9]. On the other hand, the overexpression of GnT-III (N-acetylglucosaminyltransferase III), which led to the addition of a bisecting GlcNAc to α5 integrin subunit *N*-glycans, inhibited the α5β1 integrin-mediated cell spreading and migration and subsequently down-regulated the integrin-guided signalling through FAK (focal adhesion kinase) [10]. Additionally, sialylation of the non-reducing terminus of glycans of the α5β1 integrin receptor was found to be of great importance in the adhesion of cells to fibronectin as well. Several studies pointed out that hyposialylation or desialylation of the β1 integrin subunit of the α5β1 integrin receptor enhanced the adhesiveness to fibronectin and modified cell migration and metastatic potential [11–13]. In contrast, sialic acid's removal of the α5 subunit decreased fibronectin binding [14]. These observations reveal that the sialylation patterns of both α- and β subunits can distinctly affect the cellular interactions of integrin receptors with extracellular matrices and suggest that sialic acids play a significant role in the initial carbohydrate–carbohydrate recognition and binding preceding the integrin-dependent adhesion.

Terminal sialic acid residues may also regulate CCI with other membrane-associated proteins and GSLs in the membrane. These so-called side to side (*cis*-) interactions can form complexes consisting of integrins, tetraspanins or growth factor receptors with GSLs and have been demonstrated in several co-clustering and co-precipitation studies [15]. These interactions are presumed to be caused by the many hydroxyl groups in the carbohydrate moiety, which can function to form hydrogen bonds. In addition, the negative charge of sialic acid, due to the carboxyl group, may result in supplementary repulsive or attractive forces. Along this line, it was suggested that the presence of gangliosides and sialoglycoconjugates resulted in repulsive interactions, while attractive interactions were detected between two neutral GSLs or between neutral GSLs and sialic acid containing glycoconjugates [16,17].

In our previous studies, we had demonstrated that the GSL, AsGM1 (asialo-GM1) and the α2β1 integrin receptor are important mediators of the metastatic behaviour of the bone metastatic derived C4-2B cells of the LNCaP/C4-2B prostate cancer progression model. We found that AsGM1 and the α2β1 integrin receptor were reorganized, clustered and co-localized in the bone metastatic C4-2B cells, as compared with the parental, non-invasive LNCaP cells. This complex mediated the activation of a downstream signalling pathway leading to the release of proteases, which facilitated invasion into collagen type I [18,19]. These observations raised questions on the role of sialylation, given that no complex was formed with the GM1 ganglioside, which is related to AsGM1 but contains a α2,3-linked sialic acid residue, and was further supported by the fact that altered sialylation modulates integrin function and presumably also its assembly properties [15]. Therefore the experiments described here were undertaken to (i) investigate the differences in sialylation between LNCaP and C4-2B cells; explore how these changes contributed to (ii) the metastatic behaviour of the C4-2B cells; and (iii) the complex organizational status of the α2β1 integrin receptor with AsGM1 in C4-2B cells. In this study, we show that the presence of sialic acids plays a significant role in the metastatic behaviour of the C4-2B cells. Indeed, we found a difference in the sialylation pattern between the parental, non-invasive LNCaP and the bone metastatic C4-2B cells. More specifically, an increase in α2,3-linked sialic acid residues on the α2 subunit of the integrin α2β1 receptor was observed at the cell surface of C4-2B cells, which correlated with increased gene expression of α2,3- STs, particularly *ST3GAL3*. Additionally, our data demonstrate an important functional role of the cell surface α2,3-sialylation on the α2β1 integrin receptor in the interaction and adhesion to collagen type I. These data suggest that the same α2,3-linked sialic acid residues on the integrin receptor contribute to the interaction with the carbohydrate moiety of AsGM1, resulting in complex formation of the α2β1 integrin receptor and AsGM1, in C4-2B cells, elucidating our previous data [18,19] and providing novel insights into the role of sialic acids in the organization and function of important membrane components in the invasion process.

## EXPERIMENTAL

### Antibodies and other reagents

The antibodies against the integrin α2β1 receptor, the α2 integrin subunit and AsGM1 were from EMD Millipore [18,19], while the β1 integrin antibody was from BD Biosciences and secondary ALP (alkaline phosphatase)-labelled anti-mouse and anti-rabbit antibodies were from Promega. Mouse and rabbit monoclonal IgG isotype antibodies were from Cell Signaling Technology. Biotinylated-MAA (*Maackia amurensis* agglutinin) and SNA (*Sambucus nigra* agglutinin), as well as fluorescein-labelled SNA, Fluorescein Avidin DCS and Vectashield mounting medium were obtained from Vector Laboratories. FITC-labelled- MAA and SNA were purchased from EY Laboratories. DIG (digoxigenin)-conjugated MAA and SNA, anti-DIG-labelled ALP and NBT/BCIP (Nitro Blue Tetrazolium/5-bromo-4-chloroindol-3-yl phosphate) substrate, included in the DIG Glycan Differentiation Kit, and sialidase from *Clostridium perfringens* were from Roche Diagnostics. BCA protein assay reagent kit was from ThermoFisher Scientific Inc. GM1 and AsGM1 were from Sigma.

### Cell culture

The human prostate cancer LNCaP cells and the bone metastatic derivative cell line, C4-2B, were a gift from Dr M. Bisoffi and Dr G. Thalmann (UNM, School of Medicine, NM and University of Bern, Switzerland) [20] and were grown in RPMI-medium supplemented with 5% (v/v) FBS, 100 IU/ml penicillin, 100 μg/ml streptomycin (ThermoFisher Scientific Inc.) at 37°C equilibrated with 5% (v/v) CO_2_ in humidified air.

### RNA isolation and cDNA synthesis

Total RNA from LNCaP and C4-2B cells was isolated using the NucleoSpin® RNA II (Macherey-Nagel) following the manufacturer's instructions. Isolated RNA (1–2 *μ*g) was subsequently subjected to reverse transcription in the presence of oligodeoxythymidilic acid_12−18_ primer (First-strand cDNA synthesis kit; GE Healthcare) for cDNA synthesis in a final volume of 33 μl according to the manufacturer's protocol.

### QPCR (quantitative PCR) analysis of ST transcriptional expression

The ST genes: *ST6GAL1* and *ST6GAL2*; *ST3GAL1*, *ST3GAL3*, *ST3GAL4* and *ST3GAL6* were studied in LNCaP and C4-2B cells by QPCR. SYBR® Green QPCR and its data analysis were performed using the MX4000 Multiplex QPCR System (Stratagene) equipped with Version 3.0 software. The oligonucleotides used as primers (Table 1) were obtained from Eurogentec and have been described previously [21–23]. Each 25 μl PCR reaction contained 12.5 μl Brilliant® SYBR® Green QPCR Mastermix (Stratagene), 300 nM of each primer and 2 μl of cDNA diluted 1:20. DNA amplification was performed according to the following thermal cycling profile: initial denaturation at 95°C for 10 min, 40 cycles of amplification [denaturation at 95°C for 1 min, annealing at 51 or 58°C (Table 1) for 30 s, and extension at 72°C for 1 min] and a final extension at 72°C for 5 min. The fluorescence monitoring occurred at the end of each cycle. The analysis of amplification results was performed using the MX4000 3.0 software. *C*_T_ (threshold cycle value) is defined as the number of PCR cycles where the fluorescence signal exceeds the detection threshold value. The equation *C*_T_=*a*×log(initial quantity)+*b* allowed us to determine the efficiencies of the reactions, which were determined from standard curves generated for each pair of primers using serial dilutions of cDNA from LNCaP and C4-2B cells and were found in a range of 97.3–101.5%. *C*_T_'s were also used to reveal the quantities of each ST transcript. QPCR assays were performed in triplicate in 96-well plates.

**Table 1:** Oligonucleotide primers pairs used for amplifying sialyltransferase and *HPRT* genes by QPCR

Gene sense primers (5′–>3′)	Antisense primers (3′–>5′)	*T*_m_ (°C)	Reference
*HPRT*	GACCAGTCAACAGGGGACAT	51	[21]
	CTTTTCCTGGGGTGCTTCACAA	58	
*ST6GAL1*	GGGCTCCAAACTAACCATCTC	51	[22]
	AAATCCAGGCTTTCTCACTCC		
*ST6GAL2*	ACGCTGCTGATTGACTCTTCT	51	[21]
	AATCTACTCACGGTCATACAC		
*ST3GAL1*	GGGCAGACAGCAAAGGGAA	58	[23]
	GGCCGTCACGTTAGACTCAAA		
*ST3GAL3*	CGGATGGCTTCTGGAAATCTGT	58	[22]
	TTGTGGTCCAGGACTCTTTGA		
*ST3GAL4*	CCCAAGAACATCCAGAGCCTCA	58	[23]
	CGTGGTGGGCTTCTGCTTAATC		
*ST3GAL6*	TTTTGAGGAGGATATTTGGCTAC	58	[22]
	AACAAACACTGCCTTCATTGTAC		

### Sialidase treatment

Single cells were resuspended in sodium citrate buffer (pH 6) and treated with 0.5 units/ml sialidase from *C. perfringens* (Roche) in sodium citrate buffer (pH 6) for 1 h at 37°C. After treatment, cells were washed with serum-free medium or cold PBS, for cell attachment assays or flow cytometry, respectively. For the specificity of the lectins in the lectin blot analysis, part of the membrane was treated with sialidase for 16 h at 37°C.

### Flow cytometry analysis

LNCaP and C4-2B cells were detached with 0.2% (w/v) EDTA and neutralized with cold PBS. After washing with PBS, the sialidase-treated cells and untreated cells were resuspended in PBS containing 0.1% (w/v) BSA (Sigma). Next, the treated and untreated cells were incubated with biotinylated-MAA and SNA, detecting α2,3- or α2,6-linked sialic acids, respectively, for 30 min at 4°C and was followed by 15 min Fluorescein Avidin DCS incubation at 4°C or FITC-labelled MAA and SNA for 20 min. After washing, 1×10^4^ stained cells were analysed for fluorescence using the Cell Lab Quanta SC MPL (Beckman Coulter). Stainings without the specific lectins were used as controls.

### Lectin blot/affinity analysis

LNCaP and C4-2B cell lysates were made from 80 to 90% confluent LNCaP and C4-2B cultures, using 0.5 ml lysis buffer containing 1% (v/v) Triton X-100, 1% (v/v) Nonidet P40 and the following inhibitors: aprotinin (10 μg/ml), leupeptin (10 μg/ml), PMSF (1.72 mM), NaF (1 mM), NaVO_3_ (500 μM) and Na_4_P_2_O_7_ (500 μg/ml). Aliquots of lysates, containing 25 μg of protein, were boiled for 5 min in SDS/PAGE sample buffer containing 5% (v/v) 2-mercaptoethanol, electrophoresed on 4–20% gradient or 7.5% SDS/PAGE and transferred to PVDF membranes (Immobilon-P) (Bio-Rad Laboratories). Lectin blot analysis was performed using the DIG glycan Differentiation kit (Roche) following the manufacturer's protocol. Briefly, membranes were blocked for 30 min in blocking solution, washed in TBS and stained for α2,3- or α2,6-linked sialic acids by incubating the membranes with DIG-conjugated lectins MAA (1:200) and SNA (1:1000), respectively, in TBS containing 1 mM MgCl_2_, 1 mM CaCl_2_ and 1 mM MnCl_2_ (pH 7.5) for 1 h. After washing, the membranes were incubated for 1 h with anti-DIG antibody conjugated with ALP (1:1000 in TBS), which was followed by three wash steps and staining with NBT/BCIP (1:50 in 0.1 M Tris-HCl, 0.05 M MgCl_2_ and 0.1 M NaCl at pH 9.5).

### Interaction of LNCaP and C4-2B cells with collagen type I and effect of sialidase and function-blocking antibodies

LNCaP and C4-2B cells were detached with 0.2% EDTA and washed with the serum-free medium. Next, 1×10^6^ of the sialidase-treated cells and untreated cells were resuspended in culture medium supplemented with 2% FBS at a density of 1×10^6^ cells per ml. 100 μl of sialidase-treated and untreated cell suspensions, with or without function blocking integrin α2β1 (5 μg/ml) or AsGM1 (1:500) antibodies were added to collagen type I-precoated 96-well plates (BD Biosciences), and centrifuged for 1 min at 115 ***g***. After 5, 10, 30, 60, 90 and 120 min incubation at 37°C, the plates were washed four times with PBS to remove the non-adherent cells. The adherent cells were then detected and quantified by measuring the acid phosphatase activity, through solubilization of the remaining cells with 0.2% Triton X-100 and by the addition of the substrate, PNPP (*p*-nitrophenyl phosphate; Sigma). Absorbance values of the lysates were determined on a microplate reader at 405 nm (Biotek, Synergy H1). Mouse and rabbit IgG isotype control antibodies were included to estimate the non-specific binding.

### Co-IP (immunoprecipitation) of integrin α2 and β1 subunits and co-precipitation of MAA and SNA

Cells, LNCaP and C4-2B that reached 80–90% confluency were lysed, as described under ‘Lectin blot/affinity analysis’. Lysates, containing 2000 μg protein were incubated with protein G-sepharose or avidin beads (GE Healthcare; Vector Laboratories) to preclear non-specific binding. Antibodies to integrin α2 and integrin β1 subunits (1:500) or biotinylated-MAA and SNA (1:200) were added to the collected supernatant and rotated overnight at 4°C. Next protein G-sepharose or avidin beads were added to recover the immunocomplexes and the lectin/glycoprotein complexes, respectively. The precipitates were resolved in 50 μl SDS/PAGE sample buffer and heated to 95°C for 5 min. The supernatants were electrophoresed on 4–20% or 7.5% SDS/PAGE and transferred to PVDF membranes. After transfer, the membranes were analysed and developed as described under ‘Lectin blot/affinity analysis’ or were blocked and incubated for 1 h with primary antibodies against the integrin α2 and β1 subunits for detection of sialic acids or as controls for the respective co-IPs. Subsequently, the membranes were washed, incubated with secondary anti-rabbit or anti-mouse ALP-labelled antibodies for 45 min and developed using NBT/BCIP substrate.

### CCI

#### Interaction of LNCaP and C4-2B cells with AsGM1 and effect of sialidase and function-blocking antibodies

AsGM1 was coated on the plastic surfaces of 96-well plates (TPP-US) by evaporating 50 μl ethanol solution containing 1 μg of AsGM1, dried at 30°C and blocked by 2% BSA (Sigma) in PBS. Next, 5×10^4^/100 μl sialidase-treated or untreated LNCaP and C4-2B cells, with or without function-blocking integrin α2β1 (5 μg/ml) or AsGM1 (1:500) antibodies, were seeded in each well and incubated for indicated times. The adherent cells were determined and expressed as described above under ‘Interaction with collagen type I’ as well as the influence of non-specific binding using IgG isotype control antibodies.

### Statistical analysis

Data were expressed as means±S.D., from at least three independent experiments. Data with normal distribution and homogenous variances were analysed with Student's *t* test. The criterion for significance was set at *P*<0.05. Levels were quantified using Scion Image statistical software (Scion Corporation).

## RESULTS AND DISCUSSION

Cancer accounts for one-quarter of deaths in developed, high-income countries of which 90% can be attributed to metastasis [24]. However, the mechanisms that govern cancer metastasis are not fully understood, and this reflects in the limited availability of treatments for advanced disease. Our previous studies and other recent studies indicate that there is a complex and careful interplay between the different molecules involved in cancer progression. More specifically, reorganization and clustering of membrane proteins and/or lipids and their assembly with signalling molecules, resulting in the activation of downstream signalling pathways regulating cancer cell–cell and cell–ECM adhesion, migration and invasion [15,18,19,25,26]. Such dynamic organizations and combinations are assumed to be maintained by well-defined interactions and are presumed to be affected by the glycosylation status of the functional proteins and their effect on surrounding gangliosides forming dynamic complexes [15,25,27].

The present study was undertaken to identify and address the functional significance of changes in sialylation during prostate cancer progression, using the isogenic LNCaP/C4-2B prostate cancer progression model. This model shows considerable similarities with the human progression of prostate cancer and is an ideal cell model system to study new hypotheses *in vitro* prior to proceeding to *in vivo* studies [20].

### Differences in sialylation between LNCaP and C4-2B cells

**Figure 1:**
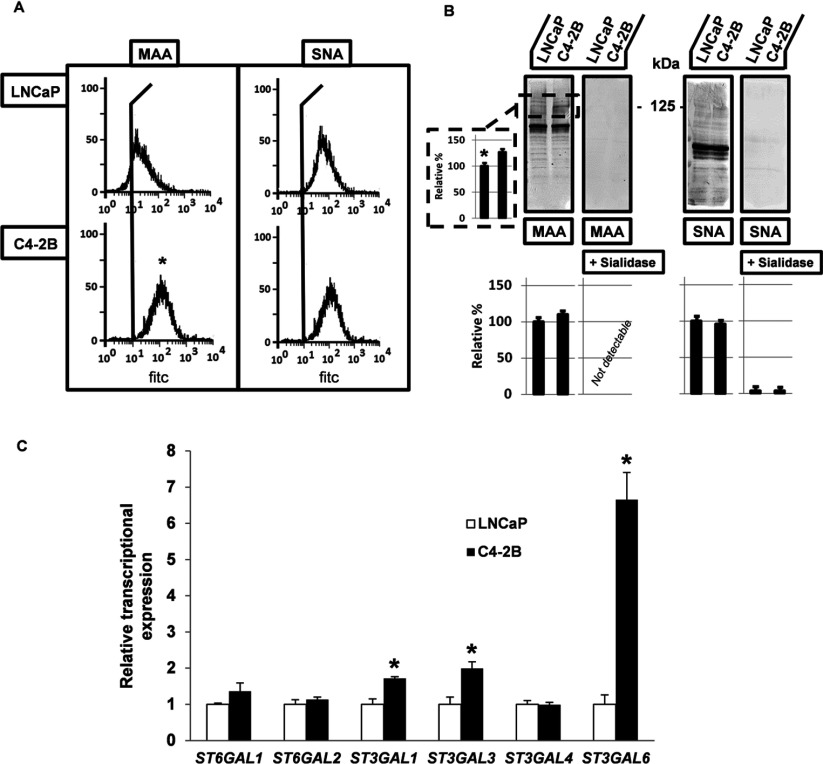
Differences in sialylation between parental, non-invasive LNCaP and bone metastatic C4-2B cells (**A**) Flow cytometric determination of increased cell surface expression levels of α2,3- (left panel) and equal expression levels of α2,6- (right panel) linked sialic acid residues in C4-2B cells, using the lectins MAA and SNA, respectively. Single cell suspensions of LNCaP and C4-2B cells were incubated with biotinylated-MAA and SNA, followed by Fluorescein Avidin DCS and analysed on the Cell Lab Quanta SC MPL, stainings without the particular lectins were used as controls. Each experiment was performed at least three times. (**B**) Lectin blot analysis for total expression levels of α2,3- and α2,6-linked sialic acids in LNCaP and C4-2B cell lysates, containing 25 μg protein. Lysates were analysed by 4–20% gradient or 7.5% SDS/PAGE, and blotted with MAA (left panel) and SNA (right panel). Sialidase treatment (0.5 U/ml sodium citrate buffer at pH 6) of the blotted membranes for 16 h at 37°C, serves as control for the presence of sialic acid residues. Scion Image densitometry analysis of bands indicating the presence of α2,3- and α2,6-linked sialic acids in both cell lines (lower panels) and evaluation of the increased density at 125 kDa in C4-2B cells (side left panel) (**C**) Transcriptional expression levels of ST genes resulting in α2,3- and α2,6-linked sialic acid residues. Expression levels were determined by QPCR, normalized against HPRT (hypoxanthine–guanine phosphoribosyltransferase) and levels present in C4-2B cells are expressed relative as compared with the expression in the parental, non-invasive LNCaP cells. Analysed and evaluated data are means±S.D. from at least three independent experiments, asterisks indicate statistical difference from parental, non-invasive LNCaP control cells (*P*<0.05).

In order to determine whether altered sialylation plays a functional role in the metastatic behaviour of C4-2B prostate cancer cells, as compared with the parental LNCaP cells [18,19], we initiated our studies by examining the differences in sialylation between the two cell lines at different levels of expression, namely cell surface, total and transcriptional expression levels (Figure 1). By using the MAA and SNA lectins, detecting α2,3- and α2,6-linked sialic acid residues, respectively, the cell surface expression pattern and total expression levels were studied. The flow cytometry results (Figure 1A) revealed an increase in α2,3-sialylation at the cell surface of the metastatic C4-2B cells as compared with the parental LNCaP cells after MAA labelling. A slight but an insignificant increase of α2,6-linked sialic acids upon labelling with SNA was also observed and as expected, the binding of both lectins was abolished upon sialidase treatment that removed the sialic acid residues at the cell surface of both the cell lines (results not shown). Lectin blot analysis showed that α2,3-sialylation was increased in total cell lysates of C4-2B cells as compared with LNCaP cells and this particularly at a molecular weight of about 125 kDa, while no other major changes were found at other molecular weights or after labelling with SNA (Figure 1B). The transcriptional expression levels of the STs responsible for α2,3- and α2,6-sialylation, including α2,3-ST genes *ST3GAL1*, *ST3GAL3*, *ST3GAL4* and *ST3GAL6* and α2,6-ST genes, *ST6GAL1* and *ST6GAL2*, were screened quantitatively by QPCR. When evaluating the transcriptional expression levels found in the metastatic C4-2B cells as compared with the control LNCaP, a 2- to 6-fold increase in expression was noticed for *ST3GAL1*, *ST3GAL3* and *ST3GAL6* and no significant differences were observed for the α2,6-ST and *ST3GAL4* genes (Figure 1C). However, it needs to be mentioned that the relative expression of the different α2,3-ST genes was quite variable with the lowest expression levels for *ST3GAL6* (*C*_T_=30), the highest for *ST3GAL1* (*C*_T_=23) and intermediate levels of *ST3GAL3* with a *C*_T_ of 27 (results not shown). Overall, these results indicate that there are no significant differences in α2,6-sialylation between LNCaP and C4-2B cells, but that there is a significant increase in cell surface and total expression of α2,3-linked sialic acid residues, which can be directly correlated with the enhanced transcriptional expression levels of particular α2,3-ST genes in the bone metastatic C4-2B cells. These findings are in line with several other studies describing enhanced levels of *ST3GAL1*, *3* and *6* genes and higher expression of α2,3-linked sialic acids in different types of cancer associated with progressed stages of the disease. Many of these studies, however, do not suggest an exclusive increase of α2,3-sialylation and usually also show an increase in α2,6-sialylation or alterations in fucosyltransferase activity, related to the synthesis of sialyl-Lewis^x^ antigens [28–32]. Although differences were observed in the transcriptional expression levels of several fucosyltransferases between the LNCaP and C4-2B cell lines, and this mainly for *FUT3* and *FUT7*, no changes in expression levels of sialyl-Lewis^x^ were observed after Western blot analysis (results not shown). Furthermore, the lectin blot analysis (Figure 1B) reveals an increased staining intensity with MAA, in the range of 125 kDa, suggesting that molecules in this molecular weight area are more α2,3-sialylated in the metastatic C4-2B cells.

### Adhesion of C4-2B cells to collagen type I via sialylation of the integrin receptor prior to the integrin-mediated adhesion

**Figure 2:**
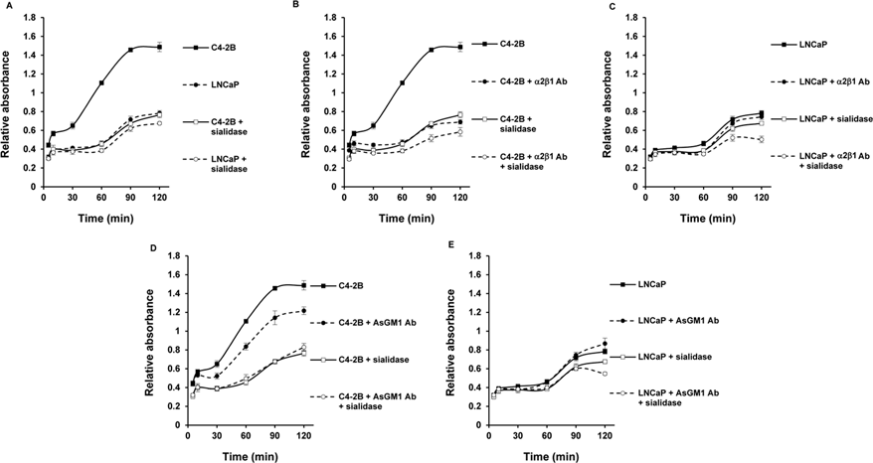
Adhesion of C4-2B cells to collagen I via sialic acids and α2β1 integrin receptor (**A**) Sialic acids expressed at the cell surface of C4-2B cells mediate adhesion to collagen I. LNCaP (dashed line, closed circles) and C4-2B (solid line, closed squares) cells and sialidase-treated (0.5 U/ml sodium citrate buffer pH 6 for 1 h at 37°C) LNCaP (dashed line, open circles) and C4-2B (solid line, open squares) at a density of 1×10^5^ cells/100 μl were seeded in a collagen type I precoated 96-well plate and incubated over a time range of 5–120 min. (**B**) Blocking the α2β1 integrin receptor (5 μg/ml) in C4-2B (dashed line, closed circles) and sialidase-treated C4-2B cells (dashed line, open circles) reduces the binding of C4-2B (solid line, closed squares) and sialidase-treated C4-2B cells (solid line, open squares) to collagen type I precoated plates as compared with (**C**) similar treatments in parental, non-invasive LNCaP control cells: untreated LNCaP cells (solid line, closed squares) and sialidase-treated LNCaP cells (solid line, open squares) and after α2β1 integrin receptor blocking of LNCaP (dashed line, closed circles) and sialidase-treated LNCaP cells (dashed line, open circles). (**D**) The function blocking of AsGM1 (1:500) affects the binding of C4-2B cells to collagen type I-coated 96-well plates (dashed line, closed circles) as evaluated against untreated bone metastatic C4-2B cells (solid line, closed squares) and is not influenced by sialidase treatment of C4-2B cells (dashed line, open circles) as compared with sialidase-treated C4-2B cells (solid line, open squares). (**E**) There are no major changes in interaction and binding of LNCaP cells (solid line, closed squares) to collagen type I, in the presence of function blocking antibody against AsGM1 (dashed line, closed circles), after sialidase treatment (solid line, open squares) or after blocking AsGM1 in sialidase-treated LNCaP cells (dashed line, open circles). At each time point, cells adhering to collagen type I were washed and numbers of cells were determined by measuring acid phosphatase activities. The interaction and adhesion to collagen type I is represented as relative absorbance, after subtracting non-specific binding. Mouse and rabbit IgG isotype control antibodies were included to estimate the non-specific binding. Six to eight wells were analysed in each experiment. All data are means±S.D. from three independent experiments (*P*<0.05).

Given the differences in sialylation between LNCaP and C4-2B cells, suggesting an altered sialylated glycol–epitope pattern at the cell surface of C4-2B cells, we studied whether these changes in sialylation influence the adhesion of LNCaP and C4-2B cells to collagen type I (Figure 2A). Based on the role of AsGM1 and α2β1 integrin receptor in adhesion to collagen type I [18,19], adhesion assays were performed in combination with the function blocking antibodies against the integrin α2β1 receptor (Figures 2B and 2C) and with AsGM1 (Figures 2D and 2E). In Figure 2(A), we demonstrate that sialylation affects the adhesion of both cell lines to collagen type I, particularly of C4-2B cells, since sialidase treatment tremendously decreased the adhesive capacity of C4-2B to collagen type I over the whole time range tested. A smaller effect was observed on the adhesive capacity of the parental LNCaP cells, which was only significant after 60 min incubation (*P*<0.05). The removal of sialic acids by sialidase treatment (0.5 unit/ml for 1 h at 37°C) was confirmed by flow cytometry (results not shown). Furthermore, function blocking of the α2β1 integrin receptor in C4-2B cells was found to significantly block the adhesion (Figure 2B), whereas no such effect was observed in the LNCaP cells in the present test conditions, which corresponds to our previously published data after 90 min incubation (Figure 2C) [18]. Treatment of sialidase-treated cells with the α2β1 integrin receptor antibody synergistically enhanced the effect on function blocking of the α2β1 receptor in C4-2B cells. Similar treatment on sialidase-treated LNCaP cells resulted in a significant decrease in adhesion to collagen type I, after 90 min (Figures 2B and 2C). Furthermore, blocking the function of AsGM1 using anti-AsGM1 antibody diminished the adhesive capacity of C4-2B cells, but not of LNCaP cells, to collagen type I. These results are also in agreement with our earlier data reported at *t*=90 min [19]. Remarkable in Figures 2(D) and 2(E) is that co-treatment of sialidase-treated cells with function-blocking antibody against AsGM1, follows exactly the same pattern as sialidase-treated C4-2B and LNCaP cells, except at *t*=120 min in LNCaP cells. In our previous study, we reported that function blocking of this integrin receptor and GSL did not result in any additional diminished adhesion to collagen type I after 90 min incubation, and also in the present study, no synergistic effect was observed by the co-antibody treatment of LNCaP and C4-2B cells, nor after sialidase treatment (results not shown). The most notable finding here is the diminished adhesiveness of sialidase-treated C4-2B cells to collagen type I, revealing that removal of sialic acid residues on C4-2B cells results in cells that interact in exactly the same manner as the parental, non-invasive LNCaP cells with collagen type I. Other significant findings are the differences between the effect of sialidase treatment or blocking of the α2β1 integrin receptor and the synergistic effect of sialidase treatment and the integrin receptor blocking in C4-2B cells. First, sialidase treatment causes a considerable decrease in binding to collagen type I in the time range from 0 to 30 min, suggesting that sialic acids play a key role in the initial interaction of C4-2B cells with collagen type I. Secondly, single treatment with the function blocking α2β1 antibody or sialidase both resulted in a substantial reduction in binding to collagen type I after 1 h incubation, demonstrating that the actual interaction of the integrin receptor with collagen occurs after the initial carbohydrate interaction. Thirdly, the combined effect of removal of sialic acid residues and blocking of the integrin receptor displays a synergistic pattern, which is possibly resulting from less initial binding and followed by preventing protein interaction with the ECM. No such effect was observed, when sialidase-treated C4-2B cells were incubated with function blocking antibodies against AsGM1, confirming the functional importance of the α2β1 integrin receptor and sialic acid residues in the interaction and binding of the bone metastatic C4-2B cells to collagen type I and together with the earlier results from the lectin blot analysis allow us to assume that the integrin receptor is more sialylated, particularly α2,3-sialylated. Furthermore, our findings also suggest that the α2β1 integrin receptor is somewhat sialylated in LNCaP cells, since desialylation and receptor blocking decreased the adhesiveness of LNCaP cells to collagen type I, to a smaller extent of course than C4-2B cells, given the cells’ lower tendency to interact with collagen type I.

### α2,3-sialylation of integrin α2 subunit in bone metastatic C4-2B cells versus α2,6-sialylation of integrin β1 subunit in LNCaP cells

Providing that our current and previous experiments demonstrated (i) a significant functional role for sialylation in adhesion of C4-2B cells to collagen type I; (ii) exclusive roles of the α2β1 integrin receptor and AsGM1 in the invasive C4-2B cells [18,19]; and (iii) enhanced MAA-staining in the lectin blot analysis of cell lysates obtained from the bone metastatic C4-2B cells around 125 kDa, a molecular weight that corresponds to that of integrin receptors, we speculated that altered sialic acid linkages on the α2β1 integrin receptor in C4-2B cells are critical for its metastatic behaviour.

**Figure 3:**
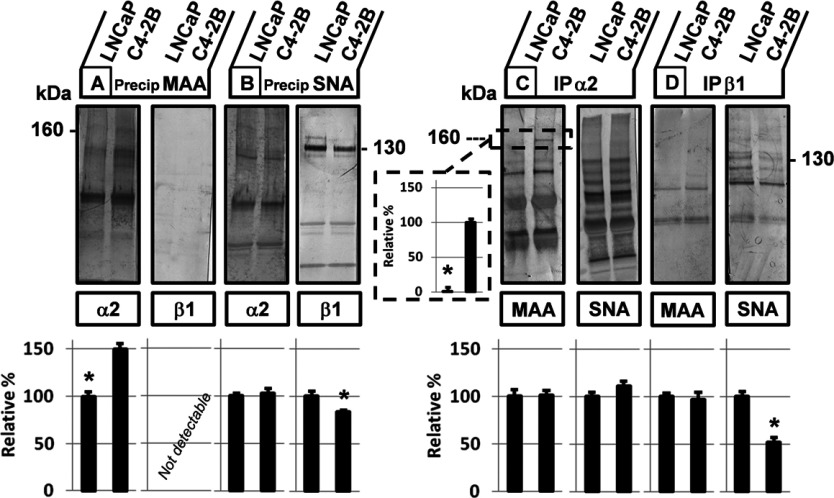
Differential sialylation of the α and β subunits of the α2β1 integrin receptor Sialic acid linkages on the subunits of the α2β1 integrin receptor were determined on cell lysates of LNCaP and C4-2B cells, containing 2000 μg protein, by a lectin affinity method using biotinylated-MAA (**A**) and SNA (**B**) (1:200) as well as co-IPs using antibodies against the (**C**) α2 integrin subunit and (**D**) β1 integrin subunit (1:500). The lectin/glycoprotein complexes and immunocomplexes were resolved in SDS/PAGE sample buffer, electrophoresed on 4–20% gradient or 7.5% SDS/PAGE, and blotted with antibodies against the α2 and β1 integrin subunits (left panel) or MAA and SNA (right panel). Lower panel: Scion Image densitometry analysis of bands indicating (**A**) left panel: an increase in α2,3-linked sialic acids on α2-integrin subunits at 160 kDa in C4-2B cells, (**B**) left panel: no significant alterations in α2,6-linked sialic acids on α2 integrin subunits, right panel: a decrease of α2,6-linked sialic acids on β1 integrin subunits (130 kDa) in C4-2B cells, (**C**) left panel: increased staining for MAA at 160 kDa in precipitates of α2 integrin subunits and no differences between LNCaP and C4-2B cells for SNA staining (right panel), (**D**) right panel: decreased staining for SNA in precipitates of β1 integrin subunits (130 kDa) in C4-2B cells. Analysed and evaluated data are means±S.D. from at least three independent experiments, asterisks indicate statistical differences between the two cell lines (*P*<0.05). Co-IPs were validated by blotting with α2 and β1 integrin subunit antibodies (results not shown).

To this end, co-IP experiments with antibodies against the α2 and β1 integrin subunits, as well as a lectin affinity approach with biotinylated-MAA and SNA, were used. As shown in Figure 3(A), MAA was able to precipitate more efficiently the α2 integrin subunits (160 kDa) in C4-2B cells and failed to precipitate β1 integrin subunits in both cell lines or at least signals were below the detection limit. SNA, on the other hand, was able to recognize β1 integrin subunits (130 kDa) more in LNCaP cells than in C4-2B cells, whereas no significant differences were observed when the membranes were blotted to detect α2 integrin subunits (Figure 3B). When the antibody against the α2 integrin subunit was used for precipitation, a significant band was detected in C4-2B cells and not in LNCaP cells when blotted with MAA, and a slightly increased band was observed for SNA in C4-2B cells as compared with the control LNCaP cells (Figure 3C). In the β1 integrin subunit precipitates (Figure 3D) following SNA staining, a markedly increased band was found in the LNCaP cells, while no signal was detected for MAA.

Taken together, the lectin affinity approach was confirmed by the results of the co-IP since both methods confirm that there is more α2,3-linked sialic acid on the α2 subunits in C4-2B cells, and that the β1 subunits are highly α2,6-sialylated, particularly in the parental LNCaP cells. Moreover, it could be concluded that LNCaP and C4-2B cells express β1 subunits that are not significantly α2,3-sialylated and that the α2-subunits express equal amounts of α2,6-linked sialic acids. Limited information is available on the glycan profile of the native transmembrane form of the α2β1 integrin receptor or the individual α2 subunit. On the other hand, the β1 subunit has been identified as a substrate for the ST ST6Gal1 [11]. The same research group published several papers linking increased α2,6-sialylation of the β1 subunit, due to increased ST6Gal1 activity, in colon adenocarcinoma and ovarian cancer cells, to tumour cell migration and invasion via enhanced β1 integrin subunit function [33,34,27]. These results are in contrast with our findings, since a decrease in α2,6-sialylation of the β1 integrin subunit was observed in the metastatic C4-2B cells. Moreover, we found the α2 subunit of the α2β1 integrin receptor to be functionally important in the metastatic behaviour [18]. Some similarities with our results are found in malignant glioma cells, expressing high levels of α2,3-linked sialic acid residues on N-linked oligosaccharides, most notably on the α3β1 integrin receptor and the absence of α2,6-linked sialic acids. These authors demonstrated that stable transfection of the *ST3GAL3* gene, further increasing the cell surface expression of α2,3-linked sialic acids, stimulated their invasiveness, while transfection of the *ST6GAL1* gene meant to replace α2,3-linked sialic acids with α2,6-linked sialic acids, abolished invasion and induced alterations in adhesion to collagen and fibronectin, leading to reduced adhesion-mediated protein tyrosine phosphorylation of FAK and this resulting from improper sialylation of the α3β1 integrin receptor. Although this study did not mention sialylation of a specific subunit, their observations and the current results point out the importance of both the linkage and expression levels of terminal sialic acid residues [35,36]. In addition, the present study demonstrates the differential expression of specific sialic acid linkages on the α and β-subunits and allows us to suggest that functional activity of the integrins can be modified as a result of different sialic acid linkages on one subunit, thereby affecting the signalling through the integrin. This assumption may also explain for the first time why the α2 subunit of the α2β1 integrin receptor, rather than the β1 subunit, was found to be responsible for the initiation of the downstream signalling via tyrosine kinases FAK and Src [18]. So far, we elucidated the sialylation patterns of both α- and β subunits and elaborated on how this influences the initial carbohydrate-dependent and subsequent integrin-dependent adhesion to collagen type I in C4-2B cells, mimicking advanced prostate cancer. An additional aim of this study was to relate the obtained glycan information to the complex organizational status of the α2β1 integrin receptor with AsGM1 in C4-2B cells [19].

### *cis*-CCI and binding of C4-2B cells to AsGM1 via sialic acids

**Figure 4:**
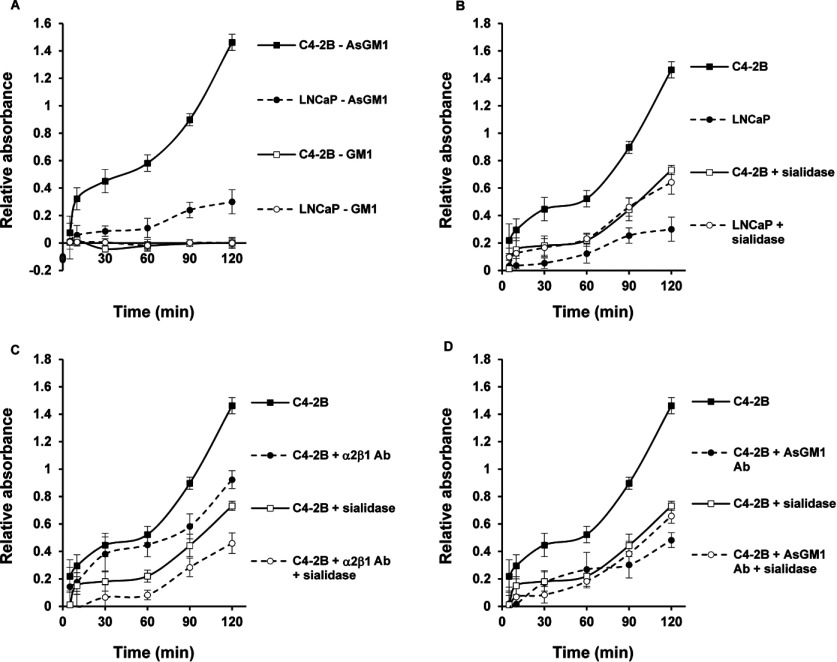
CCI of sialic acids and AsGM1 in C4-2B cells (**A**) Interaction and binding of C4-2B cells to AsGM1 (solid line, closed squares) and GM1-coatings (solid line, open squares) in comparison with interaction of LNCaP cells to AsGM1 (dashed line, closed circles) and GM1 (dashed line, open circles) (**B**) the role of sialic acids in the interaction and binding of C4-2B (solid line, closed squares) and sialidase-treated C4-2B cells (solid line, open squares) and LNCaP (dashed line, closed circles) and sialidase-treated LNCaP cells (dashed line, open circles) to AsGM1-coated layers. (**C**) The effect of removal of sialic acids (open squares and circles) or/and blocking of the α2β1 integrin receptors (dashed lines) on the interaction of C4-2B cells to AsGM1-coated layers (solid line, closed squares) and (**D**) the effect of removal of sialic acids (open squares and circles) or/and blocking of AsGM1 (dashed lines) on the interaction of C4-2B cells to AsGM1-coatings (solid line, closed squares). For all experiments, 5×10^4^ sialidase-treated (0.5 U/ml sodium citrate buffer pH 6 for 1 h at 37°C) or untreated LNCaP and C4-2B cells/100 μl, were incubated on AsGM1- or in respective experiment on GM1-coated layers for indicated times, in the presence or absence of function blocking integrin α2β1 (5 μg/ml) or AsGM1 (1:500) antibodies. Adherent cells were determined at each time point, by measuring acid phosphatase activities and results are expressed as relative absorbance, taking in account non-specific binding using IgG isotype control antibodies. Six to eight wells were analysed in each experiment. All data are means±S.D. from three independent experiments (*P*<0.05).

Clusters of the AsGM1 were found co-clustered and co-localized with the reorganized and clustered α2β1 integrin receptors in the bone metastatic derived C4-2B cells. This was shown by confocal microscopy studies and further confirmed with co-IP experiments. In contrast, no complexes were formed with the GM1 ganglioside, which is related to AsGM1 but lacks a α2,3-linked sialic acid group in its carbohydrate portion [19]. These findings suggested that the presence of sialic acids prevented the co-clustering and complex formation of these two different classes of membrane-associated molecules at the cell surface of LNCaP cells. The latter is in line with observations demonstrating that the presence of terminal sialic acids may regulate CCIs [15] and strengthens our assumption that the sialic acid group in the carbohydrate moiety of GM1 impedes the side-to-side CCI, between GM1 and the α2β1 integrin receptor, while the absence of sialic acid in AsGM1 favours such *cis*-interactions. Therefore, the possible role of sialic acid in the regulation of CCIs, as driving forces in the clustering and complex formation of several molecules of AsGM1 with α2β1 integrin receptors in C4-2B cells, was examined. In order to study these interactions, we designed a method using AsGM1-coated plastic surfaces. The idea was that the terminal sialic acids on the α2β1 integrin receptor in C4-2B cells, as was described in above results, are responsible for interaction with AsGM1. In addition, CCIs are thought to be a fast process, which can be mimicked by short incubation times and compared with prolonged incubation times, whether or not after sialidase treatment to determine the influence of sialic acids in this process. Although this method might not be the utmost technical experimental design, it was our aim to show interaction of the carbohydrate portion of AsGM1 with sialic acid residues on intact cells, displaying their specific features and properties, and thus maintaining their phenotypes as previously published [18–20]. Hence, we coated plastic surfaces of 96-well plates with various concentrations of AsGM1 and GM1, ranging from 0.01 to 10 μg, and determined that 1 μg of each GSL was the optimal concentration for binding 5×10^4^ cells (results not shown). The latter is in agreement with the previous study of Kojima et al. [16]. In Figure 4(A), we show that C4-2B cells have a greater tendency than LNCaP cells to orient themselves towards and interact with AsGM1-coated plates, and that both cell lines do not significantly adhere to GM1-coated wells. These results were expected, since we previously observed a complex formation of the α2β1 integrin and AsGM1 in C4-2B cells, and a lack thereof in LNCaP cells and the absence of similar complexes of GM1 with α2β1 integrin receptors [19]. Additionally, we found that sialidase treatment of C4-2B cells significantly decreased their capacity to interact with AsGM1, while similar treatment of the parental LNCaP cells increased the binding with AsGM1 (Figure 4B). It must be noted that sialidase treatment of LNCaP and C4-2B cells; or in other words, the removal of the cell surface sialic acid residues, leads to two cell lines that show a similar tendency to adhere to AsGM1. These results point out that sialic acids present at the cell surface of these cell lines play a crucial role in the orientation towards and interaction with AsGM1. More specifically, the presence of terminal sialic acid residues at the surface of C4-2B cells allow for attractive interactions with the neutral GSL AsGM1, whereas sialic acids on the parental LNCaP cells cause repulsive interactions. In view of our obtained data, which could mean that the increased amount of α2,3-linked sialic acids in C4-2B cells are in a more favourable conformation than the sialic acids in LNCaP cells allowing for a better interaction with the neutral carbohydrate moiety of AsGM1. Given that there are several other surface glycoproteins that can be sialylated and mediate the interaction with AsGM1; the best explanation would be that in C4-2B cells α2,3-sialylation of the α2β1 integrin receptor, particularly the α2 subunit, facilitates the CCI with AsGM1, resulting in a functionally active complex able to initiate downstream signalling events and invasion [18]. To test the latter, the metastatic C4-2B cells were treated with the function blocking antibody against the α2β1 integrin receptor. Results shown in Figure 4(C) indicate that antibody treatment did not significantly decrease the interaction and adhesion of C4-2B cells to AsGM1 within 1 h. However, a pronounced and significant α2β1 integrin-mediated inhibition, was observed after 90 and 120 min of antibody incubation. These results imply that CCIs are essential for the initial complex formation between AsGM1 and the integrin and that subsequent CPI (carbohydrate-to-protein interaction) completes the complex formation. The use of anti-α2β1 integrin on sialidase-treated C4-2B cells, further confirmed the CCI and CPI, showing decreased adhesion and interaction with AsGM1 within minutes of incubation and a pronounced synergistic effect after long-term incubation. The data in Figure 4 supports key roles for sialic acids on the α2β1 integrin receptor in metastatic C4-2B cells in the interaction and complex formation with AsGM1, confirming our previous study [19]. The synergistic effect of sialic acid removal and function blocking antibody use, may result from less initial interaction and is probably followed by a better function blocking of the integrin receptor, which may result from less sterical hindrance of the antibody recognition. When using the AsGM1 function blocking antibody, the interaction of C4-2B cells with AsGM1 was reduced, while function blocking of AsGM1 in sialidase-treated C4-2B cells had no additional effect on the interaction and adhesion to AsGM1 (Figure 4D), proving and confirming the clustering of AsGM1 [19]. This clustering likely occurs through CCI and is not significantly influenced by the presence of neighbouring sialic acid residues. The acquired data allow us to conclude that the previously detected AsGM1–α2β1 integrin complexes in metastatic C4-2B cells may result from CCIs: clustering of AsGM1 in the cell membrane may arise from *cis*-interactions between the extracellular glycan part of AsGM1 and presumably van der Waals interactions between the lipid tails, when present in the membrane. The co-localization of the clustered AsGM1 with α2β1 integrin receptors likely occurs via synergistic CCI, more specifically attractive interactions between terminal sialic acid residues and hydroxyl groups on the carbohydrate portion of AsGM1 and the formation of hydrogen bonds between the many hydroxyl groups on the carbohydrate moieties of both AsGM1 and integrin α2β1 prior to CPIs.

Overall, the present study demonstrates that differences in terminal sialic acid linkages underlie the invasive and metastatic behaviour of the C4-2B cells of the LNCaP/C4-2B progression model. In our previous studies, no changes in the expression levels were found for the cell surface molecules or for the signalling molecules mediating the downstream signalling towards the release of proteases. Instead reorganization, clustering and co-localization of cell surface molecules were found to initiate the signalling process [18,19]. The present study provides an explanation for the complex formed between a neutral GSL and a cell surface receptor, and is another example of *cis*-CCI, namely interaction between AsGM1 and α2,3-linked terminal sialic acids on glycans of α2β1 integrin receptors affecting the integrin-mediated adhesion and signalling [18,19]. Although *cis*-CCI occurs between molecules expressed within the same domain at the cell surface, *trans-*CCI arises between molecules on interfacing cell membranes [15]. Our results also suggest *trans*-CCI, given that AsGM1-coating allows interaction with AsGM1 and perhaps also involves the α2β1 integrin receptor. *Trans-*CCI was shown to be involved in cell–cell adhesion and signalling [15], and since C4-2B cells form compact aggregates on an agar layer, as compared with the scattered pattern of the non-invasive and parental LNCaP cells (results not shown), further studies are needed to elucidate the role of sialic acids in *trans*-CCI mediated by AsGM1 or other GSLs and glycoproteins present in LNCaP and C4-2B cells mediating cell adhesion and are currently in progress.

The present study fortifies the novel idea we suggested in our previous studies [18,19,26] that tumour malignancy does not necessarily arise from increased or decreased expression of genes, rather could result from a disorganization of existing molecules, held together, repulsed and influenced by carbohydrates, and demonstrates a complex interplay between lipids, proteins and carbohydrates. Although many researchers are aware of this complexity in biological processes, very few studies bring all these elements together. This idea could be a breakthrough, and provide novel targets for diagnosis and therapy. A next logical step in providing a breakthrough would be to step away from the LNCaP/C4-2B prostate cancer progression model and validate our findings in tissue from patients or TMAs (tissue microarrays), given the limitations of cancer cell lines and the alleged poor representation of the disease, like it occurs in an actual patient. Nevertheless, this model is a good reflection of what is going on in people as prostate cancer progresses, since it is associated with decreased sensitivity to androgens and leads to bone metastases [20]. The use of patient samples or TMAs to verify the significance of this study would imply that tissues are needed from different levels of aggressiveness and stages. Specifically, primary and metastatic specimens obtained from the same patient, which is extremely rare and difficult to obtain [37]. Moreover, the potentially most valuable and useful tissues would preferably be acquired from untreated patients, as treatment may easily affect the glycosylation pattern and disturb the dynamically formed complexes and subsequent signalling. While these TMAs are unbelievable resources and provide many advantages in cancer research, the usefulness in the present study is limited. Our research group is currently investigating the complex interplay of molecules in additional models of cancer progression. We plan to verify our findings in a mouse progression model system, permitting the elimination of the confounding and data compromising effects due to patient variability and treatment. This concept is currently being prepared in a proposal to cover the financial aspect of such *in vivo* studies.
